# Needle bevel geometry influences the flexural deflection magnitude in ultrasound-enhanced fine-needle biopsy

**DOI:** 10.1038/s41598-022-20161-3

**Published:** 2022-10-12

**Authors:** Saif Bunni, Heikki J. Nieminen

**Affiliations:** Medical Ultrasonics Laboratory (MEDUSA), Department of Neuroscience and Biomedical Engineering (NBE), Aalto University School of Science, 02150 Espoo, Finland

**Keywords:** Mechanical engineering, Acoustics, Biomedical engineering

## Abstract

It has been recently demonstrated that use of ultrasound increases the tissue yield in ultrasound-enhanced fine-needle aspiration biopsy (USeFNAB) as compared to conventional fine-needle aspiration biopsy (FNAB). To date, the association between bevel geometry and needle tip action has not been widely explored. In this study, we studied the needle resonance characteristics and deflection magnitude of various needle bevel geometries with varying bevel lengths. With a conventional lancet, having a 3.9 mm long bevel, the tip deflection-to-power ratio (DPR) in air and water was 220 and 105 µm/W, respectively. This was higher in comparison to an axi-symmetric tip, having a bevel length of 4 mm, which achieved a DPR of 180 and 80 µm/W in air and water, respectively. This study emphasised the importance of relationship between flexural stiffness of bevel geometry in the context of various insertion media and, thus, could provide understanding on approaches to control post-puncture cutting action by modifying the needle bevel geometry, essential for the USeFNAB application.

## Introduction

Fine-needle aspiration biopsy (FNAB) is a method employing needles to obtain a tissue sample from a suspected pathology^1–3^. It has been shown that Franseen-type tips obtain higher diagnostic yield than the conventional lancet^4^, and a Menghini tip^5^. Axi-symmetric (i.e. circumferential) bevels have also been suggested to increase the likelihood of a histo-pathologically adequate sample^6^.

During a biopsy, the needle is penetrated through the skin and layers of tissue to access suspected pathology. Recent studies suggest that ultrasonic actuation could reduce the required puncture forces into soft tissue^7–10^. Geometry of needle-bevel has been shown to influence needle-interaction forces, for example, longer bevel lengths have been shown to exhibit lower tissue-puncture forces^11^. After the needle has penetrated tissue surface i.e. post-puncture, it has been suggested that cutting forces of needle could contribute up to 75% of the total needle-tissue interaction forces^12^. In post-puncture stages, it has been demonstrated that ultrasound (US) could increase the diagnostic biopsy-yield in soft tissues^13^. Other methods with US-enhancement of biopsy of bone have been developed for sampling hard tissues^14,15^, but no results on improvement of biopsy yield were reported. It has also been established in multiple studies, that the mechanical displacement increases with increased ultrasound driving voltage^16–18^. While there are many studies concerning the axial (longitudinal) static forces in needle-tissue interaction^19,20^, there has been limited research on temporal dynamics and needle bevel geometry in ultrasound-enhanced FNAB (USeFNAB).

**Figure 1:**
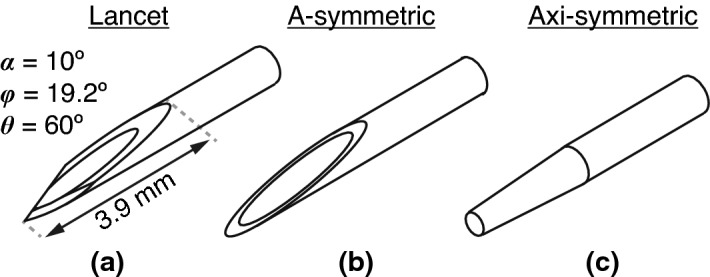
Different bevel geometries included in this study. (**a**) Lancet with specifications according to ISO 7864:2016^36^, where $\alpha$ was the primary bevel angle, $\theta$ was the secondary bevel rotation angle, and $\phi$ was the secondary bevel angle, when rotated, measured in degrees ($^\circ$). (**b**) Linear a-symmetric single step bevel (referred to as “standard” in DIN 13097:2019^37^), and (**c**) linear axi-symmetric (circumferential) single-step bevel.

The aim of this study was to investigate the role of different bevel geometries on needle tip action, in a needle flexurally-actuated at an ultrasonic frequency. More specifically, we studied in post-puncture, the influence of insertion-medium on needle tip deflection, for a conventional needle bevel (i.e. the lancet), axi-symmetric, and a-symmetric single-step bevel geometries (Fig. 1). Understanding how the needle-tip action is controlled could be beneficial in the development of USeFNAB needles for different purposes, such as selectively obtaining an aspirate or soft tissue cores.

## Methods

Our approach was to first model the change of flexural wavelength along the bevel, for a conventional lancet, axi-symmetric, and a-symmetric single-step bevel geometries. We then computed a parametric study, to investigate the effect of bevel and tube length on the transfer mechanical mobility. This was conducted in order to identify optimal lengths appropriate for fabrication of prototype needles. Informed by the simulations, prototype needles were fabricated, and their resonant behaviour was experimentally characterised, by measuring the voltage reflection coefficients and calculating the power transfer efficiency, in air, water, and ballistic gelatin 10% (w/v), from which an operational frequency was identified. Finally, the flexural-wave deflection at the needle-tip was directly measured in air and water using high-speed imaging, and the electrical power transmitted and the deflection-to-power ratio (DPR) to insertion medium, were estimated for each bevel geometry.

### Flexural wavelength and finite element model (FEM)

**Figure 2:**
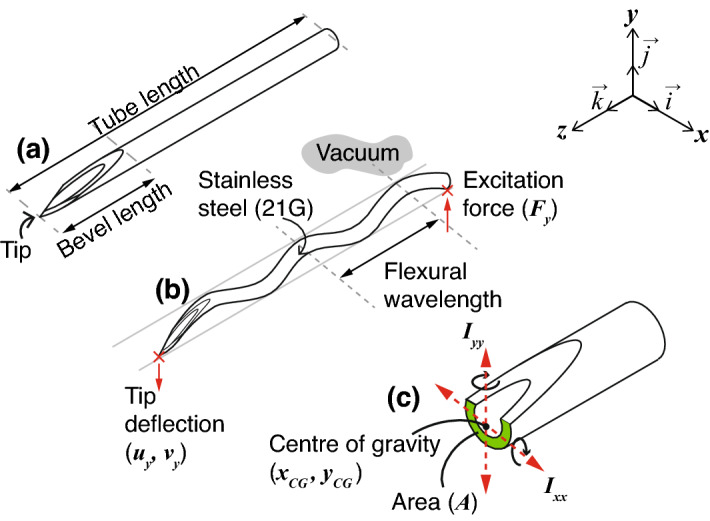
Definition of the flexural wavelength, and setup of finite element model (FEM) of needle and boundary conditions. (**a**) Definition of the bevel length (BL) and tube length (TL). (**b**) A 3-dimensional (3D) finite element model (FEM) employed a harmonic point force $\tilde{F}_y\vec {j}$ to excite the needle tube at the proximal end, a point deflection and velocity ($\tilde{u}_y\vec {j}$, $\tilde{v}_y\vec {j}$) was measured at the tip to allow a calculation of transfer mechanical mobility. $\lambda _y$ was defined as the flexural wavelength associated with the vertical force $\tilde{F}_y\vec {j}$. (**c**) Definitions of the centre of gravity, the cross-sectional area *A*, and the moments of inertia $I_{xx}$ and $I_{yy}$, around the *x* and *y* axes, respectively.

A needle tube was defined, having a tube length (TL) and bevel length (BL), as illustrated in Fig. 2a, using 21 gauge tubing (0.80 mm outer diameter, 0.49 mm inner diameter, tube wall thickness 0.155 mm, regular wall, as specified in ISO 9626:2016^21^), made of stainless steel grade 316 (*Young*’s modulus 205 $\text {GN/m}^{2}$, density 8070 kg/m$^{3}$, and *Poisson*’s ratio 0.275).

As illustrated in Fig. 2b,c, for an infinite (boundless) beam with a cross-sectional area *A*, and assuming large wavelength with respect to the cross-sectional dimension of the beam, the flexural (or bending) phase velocity $c_{EI}$ was defined^22^:

1 $$\begin{aligned} c_{EI} = \root 4 \of {EI/m'}\root 2 \of {\omega _0}, \end{aligned}$$

where *E* was the *Young*’s modulus ($\text {N/m}^{2}$), $\omega _0 = 2\pi f_0$ was the excitation angular frequency (rad/s), where $f_0$ was the linear frequency (1/s or Hz), *I* was the area moment of inertia $(\text {m}^{4})$ around the axis of interest, and $m'=\rho _0 A$ was the mass per unit length (kg/m), where $\rho _0$ was the density $(\text {kg/m}^{3})$, and *A* was the cross-sectional (*xy*-plane) area of the beam ($\text {m}^{2}$). Since the force applied in our case, was parallel to the vertical *y*-axis i.e. $\tilde{F}_y\vec {j}$, we were only concerned with the area moment of inertia around the horizontal *x*-axis i.e. $I_{xx}$, hence:

2 $$\begin{aligned} I_{xx} = \oint _A (y-y_{CG})^2 \,dA, \end{aligned}$$

where $y_{CG}$ is the *y*-coordinate of the centre of gravity of the needle tube in the *xy*-plane.

For the finite element model (FEM), a purely harmonic displacement (m) was assumed, therefore acceleration ($\text {m/s}^{2}$) was expressed as $\partial ^2 \vec {u}/\partial t^2 = -\omega ^2\vec {u}$, such as that $\vec {u}(x, y, z, t) := u_x\vec {i} + u_y\vec {j}+ u_z\vec {k}$ was a three-dimensional displacement vector defined in the spatial coordinates. Substituting the latter, the law of balance of momentum in its Lagrangian form for finite deformation^23^, was given according to its implementation in COMSOL Multiphysics software (version 5.4–5.5, COMSOL Inc., Massachusetts, USA), as:

3 $$\begin{aligned} -\rho _0\omega _0^2\vec {u}=\vec {\nabla }\cdot {\underline{\sigma }} + \vec {F_V}e^{j\phi }, \end{aligned}$$

where $\vec {\nabla }:= \frac{\partial }{\partial x}\vec {i} + \frac{\partial }{\partial y}\vec {j} + \frac{\partial }{\partial z}\vec {k}$ was the tensor divergence operator, and ${\underline{\sigma }}$ was the second Piola-Kirchhoff stress tensor (of second order, $\text {N/m}^{2}$), and $\vec {F_V}:= F_{V_x}\vec {i}+ F_{V_y}\vec {j}+ F_{V_z}\vec {k}$ was the volumetric force vector per deformed volume ($\text {N/m}^{3}$), and $e^{j\phi }$ was the phase of the volumetric force having a phase angle $\phi$ (rad). In our case, the volumetric body force was zero, and our model assumed geometrical linearity, and small purely elastic strain i.e. ${\underline{\varepsilon }}^{el} = {\underline{\varepsilon }}$, where ${\underline{\varepsilon }}^{el}$ and ${\underline{\varepsilon }}$ were the elastic and total strains, respectively (of second order, dimensionless). The Constitutive *Hookean* isotropic elasticity tensor $\underline{\underline{C}}$ was defined using the *Young*’s modulus *E* ($\text {N/m}^{2}$) and the *Poisson*’s ratio *v*, so that $\underline{\underline{C}}:=\underline{\underline{C}}(E,v)$ (of fourth order). Therefore the calculation of stress becomes ${\underline{\sigma }} := \underline{\underline{C}}:{\underline{\varepsilon }}$.

Computation was done with 10-node tetrahedral elements with element size of $\le$ 8 µm. The needle was simulated in a vacuum, and a magnitude of transfer mechanical mobility (m s^−1^ N^−1^) was defined as $|\tilde{Y}_{v_yF_y}|= |\tilde{v}_y\vec {j}|/|\tilde{F}_y\vec {j}|$^24^, where $\tilde{v}_y\vec {j}$ was the the output complex velocity at the tip, and $\tilde{F}_y\vec {j}$ was the complex driving force located at the proximal end of the tube, as illustrated in Fig. 2b. Transfer mechanical mobility was expressed in decibels (dB), using the maximum as a reference, i.e. $20\log _{10} (|\tilde{Y}|/ |\tilde{Y}_{max}|)$. All FEM studies were conducted at 29.75 kHz.

### Fabrication of needle constructs

**Figure 3:**
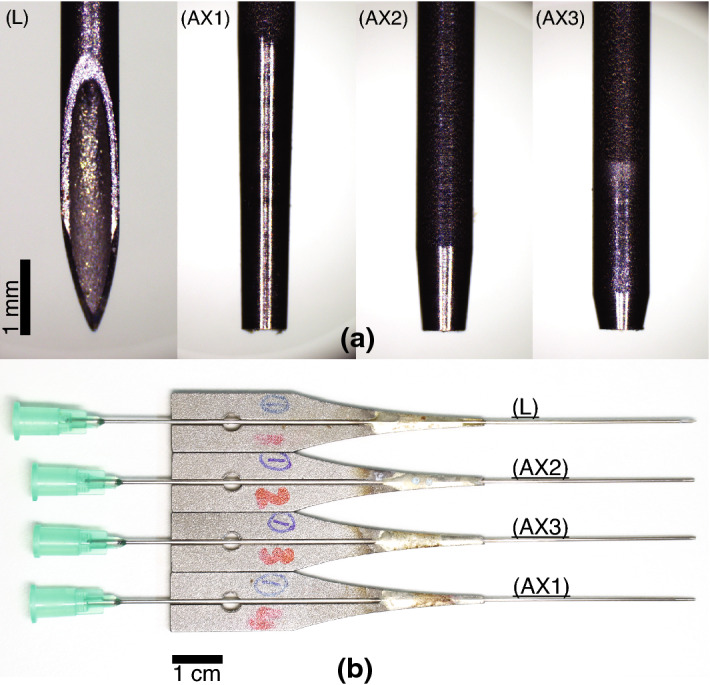
Characterisation was done for four needle tip types (photographed), a commercially-available lancet (L), and three fabricated axi-symmetric single-step bevels (AX1–3), with bevel lengths (BL) of 4, 1.2, and 0.5 mm, respectively. (**a**) a close-up side-view of the fabricated needle tips. (**b**) Top view of the four needles, soldered to a 3D-printed waveguide, which were then attached to a Langevin transducer via a M4 bolt.

The needle constructs (Fig. 3) consisted of a conventional 21 gauge hypodermic needle (catalogue number: 4665643, Sterican$^\circledR$, outer diameter 0.8 mm, length 120 mm, stainless chromium nickel steel AISI type 304 grade, B. Braun Melsungen AG, Melsungen, Germany) fitted with a Luer Lock plastic hub made of polypropylene at the proximal end, and modified accordingly at the tip. Needle tubes were soldered to waveguides, as shown in Fig. 3b. The waveguides were 3D printed with stainless steel (EOS Stainless Steel 316L in EOS M 290 3D Printer, 3D Formtech Oy, Jyväskylä, Finland), then fastened via a M4 bolt to a Langevin transducer. The Langevin transducer consisted of 8 piezo ring elements, loaded by two masses at either end.

Three axi-symmetrically bevelled tips were fabricated (Fig. 3) (TAs Machine Tools Oy) with bevel lengths (BL, as defined in Fig. 2a) of 4.0, 1.2 and 0.5 mm, corresponding to bevel angles (BA) of $\approx$ 2$^\circ$, 7$^\circ$, and 18$^\circ$, respectively. The masses of waveguides and needles were 3.4 ± 0.017 g (mean ± s.d., *n* = 4) for bevels L and AX1–3, respectively (Quintix$^\circledR$ 224 Design 2, Sartorius AG, Göttingen, Germany). The total lengths from needle tip to the end of the plastic hub were 13.7, 13.3, 13.3, 13.3 cm, for bevels L and AX1–3 in Fig. 3b, respectively.

For all needle constructs, the length from needle tip to the tip of the waveguide (i.e. soldering region), was 4.3 cm, and the needle tube was orientated so that bevel planes faced upwards (i.e. parallel to the *y*-axis), as in (Fig. 2).

### Modal analysis

A custom-script in MATLAB (R2019a, The MathWorks Inc., Massachusetts, USA), running on a computer (Latitude 7490, Dell Inc., Texas, USA), was used to generate a linear sine-sweep from 25 to 35 kHz for duration of 7 s, which was converted to an analogue signal via a digital-to-analogue (DA) converter (Analog Discovery 2, Digilent Inc., Washington, USA). The analogue signal $V_0$ (0.5 V_pk-pk_) was then amplified using a custom-made radio frequency (RF) amplifier (Mariachi Oy, Turku, Finland). The incident amplified voltage ${V_I}$ was output from the RF amplifier at an output impedance of 50 $\Omega$, to the transformer built into the needle construct, which had an input impedance of 50 $\Omega$. The Langevin transducer (back and front mass-loaded sandwich piezoelectric transducer) was used to generate the mechanical wave. The custom-made RF amplifier was equipped with a dual-channel standing-wave power ratio (SWR) meter, which allowed both the incident ${V_I}$ and reflected amplified voltages $V_R$ to be recorded via the analogue-digital (AD) converters (Analog Discovery 2) at sampling frequency of 300 kHz. The excitation signal was amplitude modulated at the beginning and end to prevent signal transients overloading the amplifier’s input.

**Figure 4:**
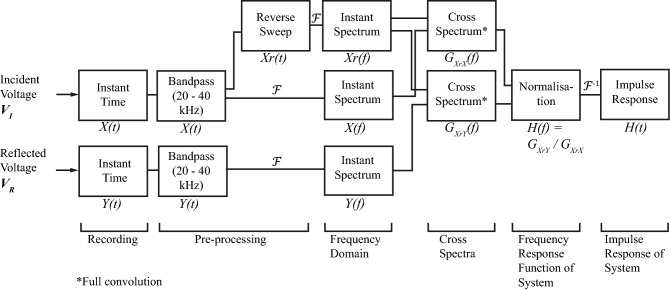
Measurement method of the frequency response functions (FRFs) of needle constructs. Swept-sine dual-channel measurement^25,38^ was used to obtain frequency response functions $\tilde{H}(f)$ and its impulse responses *H*(*t*). ${\mathcal {F}}$ and ${\mathcal {F}}^{-1}$ denote a digital truncated Fourier transform operation and its inverse, respectively. $\tilde{G}(f)$ denotes multiplication of two signals in the frequency domain, e.g. $\tilde{G}_{XrX}$ means a multiplication of the reverse sweep $\tilde{X}r(f)$ and the incident voltage $\tilde{X}(f)$ signals, respectively.

Using a custom script implemented in MATLAB, frequency response functions (FRFs) i.e. $\tilde{H}(f)$, were estimated offline using a swept-sine dual channel measurement technique^25^ (Fig. 4), which assumed a linear time-invariant system. In addition, a bandpass filter having passband between 20 and 40 kHz was applied to remove any unwanted frequencies from signal. In reference to transmission line theory, $\tilde{H}(f)$ in this case was equivalent to the voltage reflection coefficient i.e. $\rho _{V} \equiv {V_R}/{V_I}$^26^. Since the amplifier output impedance $Z_0$ was matched to input impedance of the transformer built-in with the transducer, the electrical power reflection coefficient ${P_R}/{P_I}$ was reduced to ${V_R}^2/{V_I}^2$ i.e. $|\rho _{V}|^2$. In the case when absolute values of electrical power were needed, the incident $P_I$ and reflected $P_R$ powers (W) were calculated by taking the root-mean-square (r.m.s.) of the corresponding voltages, such as that for a sinusoidally-excited transmission line, $P = {V}^2/(2Z_0)$^26^, where $Z_0$ was 50 $\Omega$. The electrical power transmitted to the load $P_T$ (i.e. to the insertion medium) could be calculated as $|P_I - P_R |$ (W, r.m.s.), and the power transfer efficiency (PTE) could be defined and given as a percentage (%), so that^27^:

4 $$\begin{aligned} \text {PTE} = \frac{P_T}{P_I} = \frac{|P_I - P_R |}{P_I} = (1 - |\rho _{V}|^2)*100. \end{aligned}$$

The FRFs were then used to estimate the modal frequencies $f_{1-3}$ (kHz) of the needle construct, and their corresponding power transfer efficiencies, $\text {PTE}_{1{-}3}$. The full-width at half-maxima ($\text {FWHM}_{1{-}3}$, Hz) were estimated directly from $\text {PTE}_{1{-}3}$, obtained from the one-sided linear frequency spectra at modal frequencies $f_{1-3}$ described in Table 1.

**Table 1:** ﻿Three modal regions of needle constructs L, and AX1–3.

Modal region	Frequency (kHz)	Description
1	$25 \le f_1 \le 27.5$	Low modal region
2	$27.5 < f_2 \le 30$	Middle modal region
3	$30 < f_3 \le 33$	High modal region

### Needle deflection measurement

**Figure 5:**
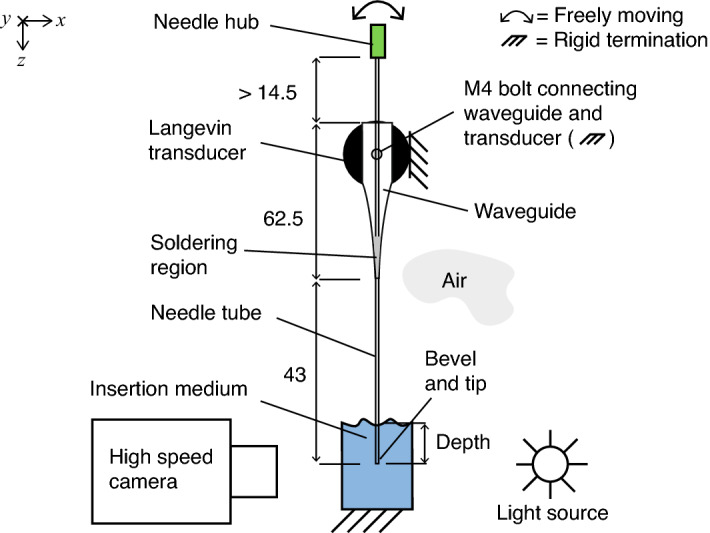
Front-view of experimental setup. Depth was measured from surface of medium. The needle construct was clamped and mounted on a motorised translation stage. A high speed camera with a high-magnification lens (5$\times$) was used to measure the deflection of bevel-tip. All dimensions are given in mm.

As shown in Fig. 5, a high speed camera (Phantom V1612, Vision Research Inc., New Jersey, USA), fitted with a macro lens (MP-E 65 mm, $f$/2.8, 1–5$\times$, Canon Inc., Tokyo, Japan), was used to record the deflection of the needle tip undergoing flexural excitation (single frequency, continuous sinusoid) at frequencies 27.5–30 kHz. In order to produce shadowgraphs, a cooled high-intensity white LED element was placed behind the needle bevel (catalogue number: 4052899910881, White Led, 3000 K, 4150 lm, Osram Opto Semiconductors GmbH, Regensburg, Germany).

**Figure 6:**
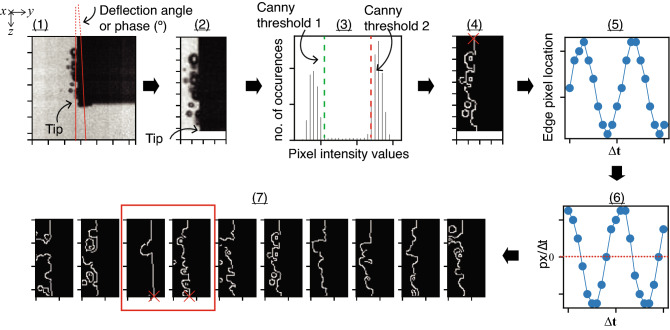
Needle tip deflection was measured using a sequence of frames captured from high-speed camera at 310 kHz, using a 7-step algorithm (1–7), involving cropping (1–2), Canny edge detection (3–4), computation of edge pixel location (5) and its time derivative (6), and finally measuring the peak-to-peak deflection at tip from a visually inspected frame pair (7).

For each needle bevel type, we recorded 300 high speed camera frames, measuring 128 $\times$ 128 pixels with a spatial resolution of 1/180 mm ($\approx$ 5 µm) per pixel, and a time resolution of 310 000 frames per second. As outlined in Fig. 6, each frame (1) was cropped (2) so that needle tip was located in the last row (bottom) of frame, then the histogram of the image was computed (3), so that Canny thresholds 1 and 2 could be determined. Then Canny edge detection^28^ with a 3 $\times$ 3 Sobel operator was applied (4), and the location was computed for a cavitation-free bevel-edge pixel (marked $\mathbf {\times }$) for all 300 time steps. To determine the peak-to-peak deflection at the tip, the derivative (using a central difference algorithm) was calculated (6), and the frames containing the local extrema (i.e. peaks) of deflection were identified (7). Following a visual inspection for cavitation-free edges, a frame pair (or two frames that are half of the time period apart) was chosen (7), and the deflection at the tip was measured (marked $\mathbf {\times }$). The above was implemented in Python (v3.8, Python Software Foundation, python.org), utilising OpenCV’s Canny edge detection algorithm (v4.5.1, Open Source Computer Vision Library, opencv.org). Finally, the deflection-to-power ratio (DPR, µm/W), was calculated as the ratio of the peak-to-peak deflection over the transmitted electrical power $P_T$ (W, r.m.s.).

### Insertion media

Measurements were done in air (22.4–22.9 °C), deionised water (20.8–21.5 °C), and aqueous ballistic gelatin 10% (w/v) (19.7–23.0 °C, $\text {Honeywell}^{\text {TM}}$ $\text {Fluka}^{\text {TM}}$ Gelatin from bovine and porcine bones, for ballistic analysis type I, Honeywell International Inc., North Carolina, USA). Temperature was measured using a thermocouple type-K amplifier (AD595, Analog Devices Inc., Massachusetts, USA), coupled with a type-K thermocouple (Fluke 80PK-1 Bead Probe no. 3648 type-K, Fluke Corporation, Washington, USA). Depth was measured from the surface of medium (set as the origin of *z*-axis), using a vertical *z*-axis motorised translation stage (8MT50-100BS1-XYZ, Standa Ltd., Vilnius, Lithuania) with resolution of 5 µm per step.

### Statistical analysis

Since the sample size was small (*n* = 5), and normality could not be assumed, a two-sample, two-sided, Wilcoxon rank sum test was used (R, v4.0.3, R Foundation for Statistical Computing, r-project.org), to compare the tip-deflection magnitudes of the different needle bevels. 3 comparisons were done for each bevel, so a *Bonferroni*-correction was applied, and the adjusted significance level was 0.017, at 5% error rate.

## Results

### Flexural wavelength and transfer mechanical mobility

**Figure 7:**
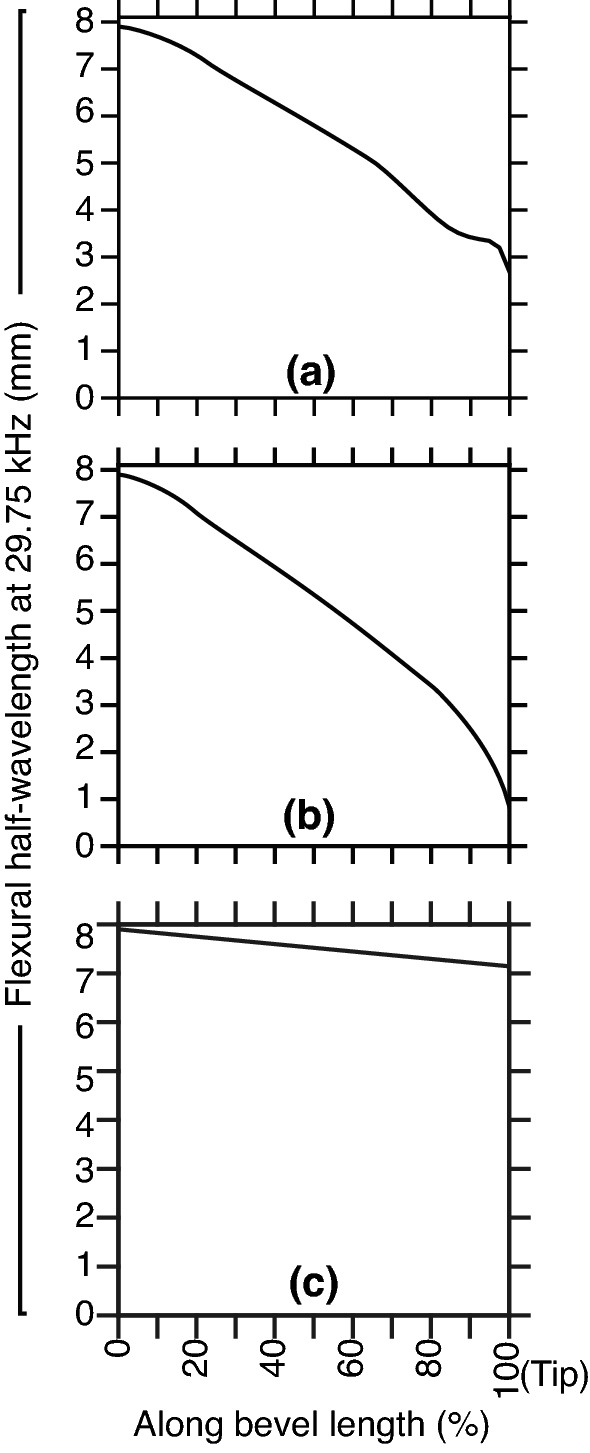
FEM studies at 29.75 kHz and application of Eq. (1) in calculation of the variation of flexural half-wavelength ($\lambda _y/2$) for the lancet (**a**), a-symmetric (**b**) and axi-symmetric (**c**) bevel geometries (as introduced in Fig. 1a,b,c). Mean $\lambda _y/2$ was 5.65, 5.17, and 7.52 mm for the lancet, a-symmetric, and axi-symmetric bevels, respectively. Note the tip thickness of a-symmetric and axi-symmetric bevels was limited to $\approx$ 50 µm.

The following refers to Fig. 7. At 29.75 kHz, the flexural half-wavelength ($\lambda _y/2$) for the 21 gauge needle tubing was $\approx$ 8 mm. The flexural wavelength decreased along bevel when approaching the tip. At the tip, $\lambda _y/2$ was $\approx$ 3, 1, and 7 mm for the conventional lancet (a), a-symmetric (b), and axi-symmetric (c) single step bevels, respectively. Consequently this meant the range of the variation was $\approx$ 5 mm for the lancet (owing to the two lancet planes generating a single sharp point^29,30^), 7 mm for the a-symmetric bevel, and 1 mm for the axi-symmetric bevel (where the centre of gravity stayed constant, so effectively only tube wall thickness varied along bevel).

**Figure 8:**
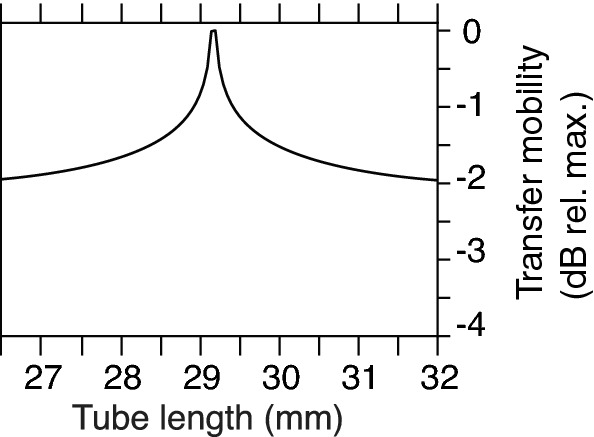
The transfer mobility for the lancet at 29.75 kHz. The needle tube was excited flexurally at 29.75 kHz, and vibration was measured at the tip and presented as the magnitude of the transfer mechanical mobility (dB relative to maximum), for TLs 26.5–29.5 mm (step size 0.1 mm).

**Figure 9:**
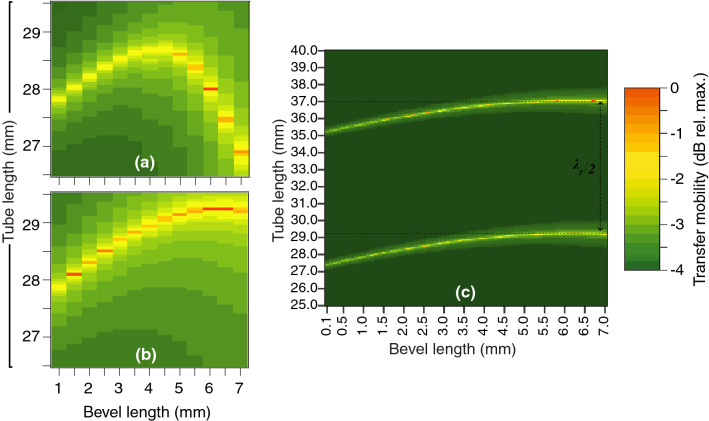
FEM parametric studies at 29.75 kHz, revealed that the transfer mobility for axi-symmetric tip was less influenced by the change in tube length than its a-symmetric counterpart. Bevel length (BL) *versus* tube length (TL) studies for a-symmetric (**a**) and axi-symmetric (**b**,**c**) bevel geometries, in a frequency domain study employing FEM (boundary conditions as in Fig. 2). (**a**,**b**) TLs range was 26.5–29.5 mm (step size 0.1 mm) and BLs 1–7 mm (step size 0.5 mm). (**c**) An extended axi-symmetric bevel study included TLs 25–40 mm (step size 0.05 mm) and BLs 0.1–7 mm (step size 0.1 mm), which revealed the $\lambda _y/2$ relationship required to satisfy the freely-moving boundary condition at the tip.

Peaks of the mobility $|\tilde{Y}_{v_yF_y}|$ indicated optimal tube length (TL) and bevel length (BL) combinations (Figs. 8, 9). For the conventional lancet, since its dimensions were fixed, the optimal TL was $\approx$ 29.1 mm (Fig. 8). For the a-symmetric and axi-symmetric bevels (Fig. 9a,b, respectively), FEM studies included BLs 1 to 7 mm, so the optimal TLs varied from 26.9 to 28.7 mm (range 1.8 mm) and 27.9 to 29.2 mm (range 1.3 mm), respectively. For the a-symmetric bevel (Fig. 9a), optimal TLs increased linearly reaching a plateau at a BL of 4 mm, then steeply declined from BLs 5 to 7 mm. For the axi-symmetric bevel (Fig. 9b), optimal TLs increased linearly with longer BLs, and eventually plateaued at BL of $\approx$ 6 to 7 mm. An extended study of axi-symmetric bevel (Fig. 9c), showed another set of optimal TLs at $\approx$ 35.1–37.1 mm. The two sets of optimal TLs were separated by a distance of $\approx$ 8 mm (equivalent to $\lambda _y/2$), for all BLs.

### Modal behaviour

**Figure 10:**
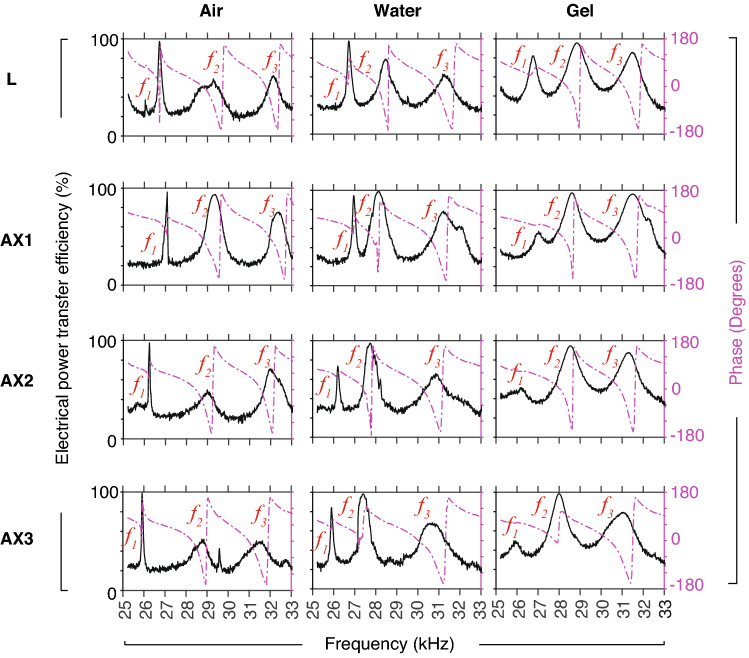
Typical recorded magnitudes of the instantaneous power transfer efficiency (PTE) obtained using swept-sine excitation, for the lancet (L) and axi-symmetric bevels AX1–3, in air, water, and and gelatin, at depth of 20 mm. One-sided spectra are shown. Measured FRFs (sampling frequency 300 kHz) were low-pass filtered, then downsampled by a factor of 200, for the purpose of modal analysis. The signal-to-noise ratio was $\le$ 45 dB. Phase (dashed purple line) of PTE is shown in degrees ($^{\circ }$).

**Figure 11:**
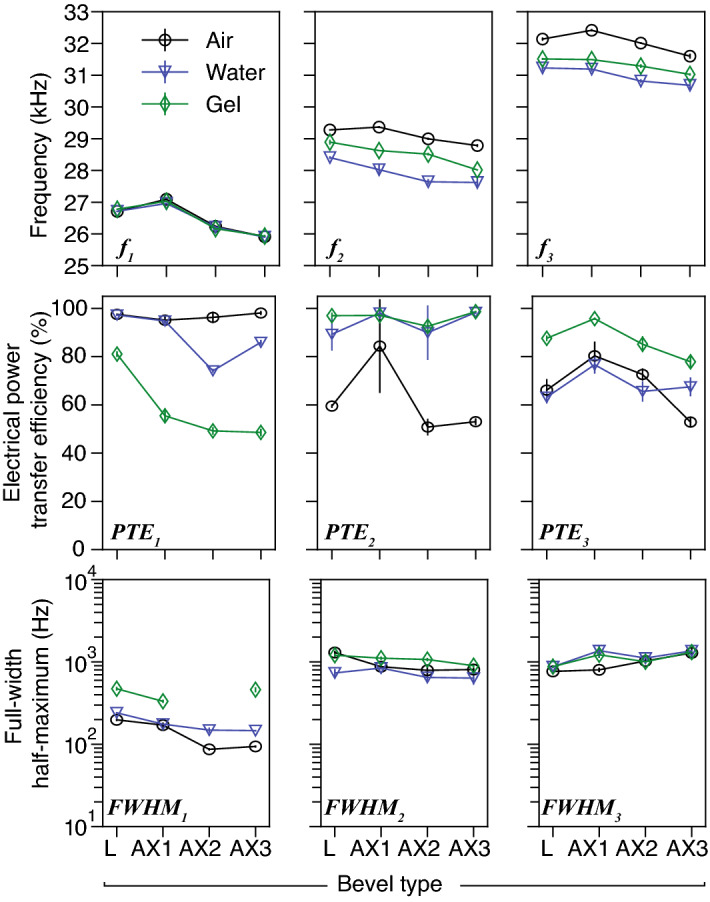
Analysis of the modal responses shown in Fig. 10 (mean ± s.d., *n* = 5), for bevels L and AX1–3, in air, water and gelatin 10% (depth 20 mm), featuring (**top**) three modal regions (low, middle and high), and their corresponding modal frequencies $f_{1-3}$ (kHz), (**middle**) power efficiency $\text {PTE}_{1{-}3}$ calculated using Eq. (4), and (**bottom**) the full-width at half-maximum measurements $\text {FWHM}_{1{-}3}$ (Hz), respectively. Note that the measurement of bandwidth was omitted, when low PTE was recorded, i.e. in the case of bevel AX2, $\text {FWHM}_{1}$. Mode $f_2$ was considered to be the most appropriate for comparing deflection of bevels, since it exhibited the highest levels of power transfer efficiency ($\text {PTE}_{2}$), which were as high as 99%.

The needle construct exhibited three natural frequencies $f_{1-3}$, which were categorised into low, middle and high modal regions, as summarised in Table 1. The magnitudes of PTE were recorded as in Fig. 10, and then analysed in Fig. 11. The following gives overview of the findings for each modal region:

- *1st modal region*: $f_1$ did not vary greatly with type of insertion medium, but varied with changing bevel geometry. $f_1$ decreased with decreasing bevel length (27.1, 26.2, and 25.9 kHz for AX1–3, in air, respectively). The region-averages of $\text {PTE}_{1}$ and $\text {FWHM}_{1}$ were $\approx$ 81% and 230 Hz, respectively. $\text {FWHM}_{1}$ was highest in gelatin for the Lancet (L, 473 Hz). Note it was not possible to estimate $\text {FWHM}_{1}$ for AX2 in gelatin, due to low recorded magnitudes of FRF.
- *2nd modal region:* $f_2$ varied with type of insertion medium and bevel. In air, water, and gelatin, averages of $f_2$ were 29.1, 27.9, and 28.5 kHz, respectively. This modal region also exhibited PTE as high as 99%, which was the highest among all measurement groups, with a region-average of 84%. The region-average of $\text {FWHM}_{2}$ was $\approx$ 910 Hz.
- *3rd modal region:* $f_3$ frequencies varied with type of insertion medium and bevel. In air, water, and gelatin, the average values of $f_3$ were 32.0, 31.0, and 31.3 kHz, respectively. The region-average of $\text {PTE}_{3}$ was $\approx$ 74%, which was the lowest among all regions. The region-average of $\text {FWHM}_{3}$ was $\approx$ 1085 Hz, which was higher than 1st and 2nd regions.

### Measured deflection

**Figure 12:**
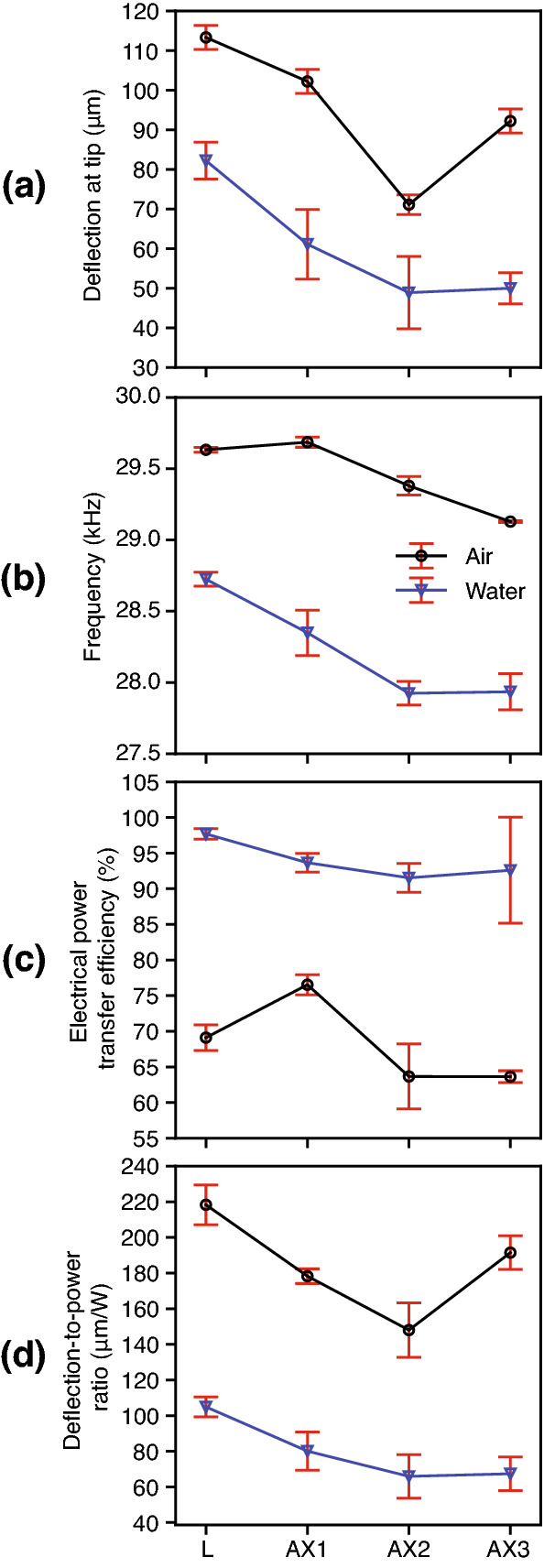
Measured flexural-deflection magnitudes of needle tip (mean ± s.d., *n* = 5) of bevels L and AX1–3 in air and water (20 mm depth), revealed the effects of changing bevel geometry. Measurements were obtained using continuous single-frequency sinusoid excitation. (**a**) Peak-to-peak deflection ($u_y\vec {j}$) at the tip-point, measured at (**b**) their respective modal frequencies $f_2$. (**c**) The electrical power transfer efficiency (PTE, r.m.s., %) as in Eq. (4), and (**d**) the deflection-to-power ratio (DPR, µm/W), which was calculated as the ratio of the peak-to-peak deflection over the transmitted electrical power $P_T$ (W, r.m.s.).

**Figure 13:**
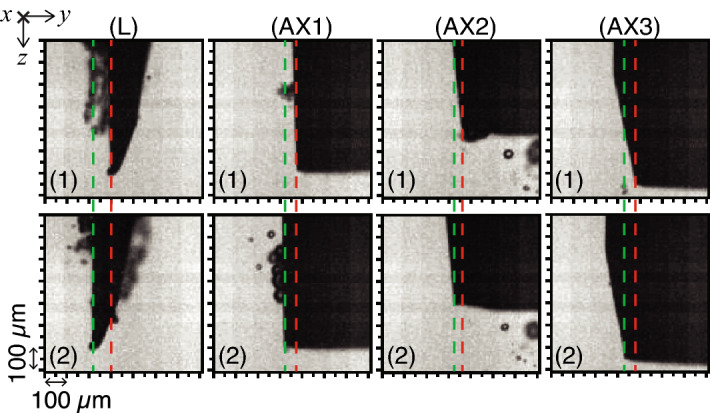
Typical high-speed camera shadowgraphs showing the peak-to-peak tip deflection (green and red dashed lines) for the lancet (L) and axi-symmetric tips (AX1–3), in water (depth 20 mm), during a half-cycle, at excitation frequency $f_2$ (sampling frequency 310 kHz). Captured greyscale images measured 128 × 128 pixels and the pixel size was $\approx$ 5 µm. A video can be found in Supplementary Information.

The following refers to Fig. 12 and Table 2. The lancet (L) deflected the most (with high significance to all tips, $p<$ 0.017) in both air and water (Fig. 12a), achieving the highest DPR (up to 220 µm/W in air). In air, AX1 which had higher BL, deflected higher than AX2–3 (with significance, $p<$ 0.017), while AX3 (which had lowest BL) deflected more than AX2 with a DPR of 190 µm/W. In water at 20 mm, no significant differences ($p>$ 0.017) were found in deflection and PTE for AX1–3. PTE levels were overall higher in water (90.2–98.4%) than air (56–77.5%) (Fig. 12c), noting cavitation events were clearly present in water during experimentation (Fig. 13, also see Supplementary Information).

**Table 2:** Was there significant difference in tip-deflection between the lancet (L) and the axi-symmetric bevels (AX1–3)?$^{\text {a}}$.

Test-pair	L-AX1	L-AX2	L-AX3	AX1–AX2	AX1–AX3	AX2–AX3
**Tests for deflection at tip (**${\varvec{u}}_{\varvec{y}}\vec {\varvec{j}}$**, Fig.** 12**a)**						
Air	0.010	0.009	0.010	0.009	0.010	0.009
Water	0.012	0.011	0.011	**0.087**	**0.052**	**0.821**
**Tests for electrical power transfer efficiency (PTE, Fig.** 12**c)**						
Air	0.008	0.016	0.008	0.008	0.008	**0.310**
Water	0.010	0.010	**0.310**	**0.095**	**0.691**	**0.691**

^a^Each pair of bevels (e.g. L *vs.* AX1) were tested using a two-sample, two-sided, Wilcoxon rank sum test, *n* = 5, 5% error rate, significance level was 0.017 (*Bonferroni*-corrected). Bolded entries indicate no statistical significance found between bevel pair﻿ (i.e. *p* $\ge$ 0.017).

## Discussion

To summarise, we modelled the change of the flexural wavelength (Fig. 7), and computed the transfer mechanical mobility for a combination of tube and bevel lengths (Figs. 8, 9), for a conventional lancet, a-symmetric and axi-symmetric bevel geometries. Based on the latter, we estimated an optimal distance of 43 mm (or $\approx$ 2.75$\lambda _y$ at 29.75 kHz) from tip to soldering region, as illustrated in Fig. 5, and fabricated accordingly three axi-symmetric bevels with varying bevel lengths. We then characterised their frequency behaviour in comparison to the conventional lancet, in air, water, and ballistic gelatin 10% (w/v) (Figs. 10, 11), and identified the mode most appropriate for comparing deflection of bevels. Finally, we measured the flexural-wave deflection at the needle tip in air and at 20 mm depth in water, and quantified the electrical power transfer efficiency to insertion medium (PTE, %) and the deflection-to-power ratio (DPR, µm/W) for each bevel type (Fig. 12).

The results show that the needle-bevel geometry affects the deflection amplitude at the needle-tip. The lancet achieved the highest deflection, as well as the highest DPR, in comparison to axi-symmetric bevels, which on average deflected less (Fig. 12). The axi-symmetric 4 mm bevel (AX1) having the longest bevel length, achieved statistically significant highest deflection in air ($p < 0.017$, Table 2), in comparison to other axi-symmetric needles (AX2–3), but no significant differences were observed, when the needle was placed in water. Therefore, in terms of the peak deflection at the tip, there was no clear benefit of having longer bevel lengths. Considering this, the results suggest that the bevel geometries investigated in this study have a greater effect on deflection amplitudes than the bevel length. This may be associated with the flexural stiffness, depending e.g. on the overall thickness of the flexurally-bending material and needle structure.

In the experimental studies, the magnitude of the reflected flexural waves were affected by the boundary conditions at the needle-tip. When the needle-tip was inserted in water and gelatin, the average of $\text {PTE}_{2}$ was $\approx$ 95%, compared to an average of 73% and 77% for $\text {PTE}_{1}$ and $\text {PTE}_{3}$, respectively (Fig. 11). This suggested that the greatest transmission of acoustic energy into the embedding medium, i.e. water or gelatin, occurred at $f_2$. Similar behaviour was observed in a previous study^31^ with a simpler device construct at 41–43 kHz, where the authors showed the voltage reflection coefficient related to the mechanical modulus of insertion medium. Penetration depth^32^ and mechanical properties of tissue provide a mechanical load on the needle, and hence are expected to affect resonant behavior of USeFNAB. Therefore, resonance tracking algorithms e.g.^17,18,33^, could be utilised to optimise the delivered acoustic power through the needle.

The simulation study of flexural wavelengths (Fig. 7) revealed the axi-symmetric had higher structural rigidity at the tip (i.e. higher flexural stiffness), than both the lancet and a-symmetric bevels. Deduced from (1), and utilising the known velocity-frequency relationship, we estimated the flexural stiffnesses at the tip to be $\approx$ 200, 20, and 1500 MPa for the lancet, a-symmetric, and axi-symmetric bevels, respectively. This corresponded to $\lambda _y$ of $\approx$ 5.3, 1.7, and 14.2 mm at 29.75 kHz, respectively (Fig. 7a–c). Considering clinical safety during USeFNAB procedures, the influence of geometry on structural stiffness^34^ of bevel needs to be assessed.

The bevel *vs.* tube length parametric study (Fig. 9), revealed that the range of optimal TLs was higher for the a-symmetric (1.8 mm), than axi-symmetric bevel (1.3 mm). In addition, the mobilities plateaued at $\approx$ 4 to 4.5 mm and at 6 to 7 mm, for a-symmetric and axi-symmetric bevels, respectively (Fig. 9a,b). The practical relevance of this finding translates to manufacturing tolerances, e.g. a lower range of optimal TLs may mean a higher precision for the lengths is required. Meanwhile, the plateaus in mobility provide greater tolerance for selecting bevel lengths at a given frequency, without significantly affecting mobility.

The study included the following limitations. Measuring needle deflection directly using edge detection and high-speed imaging (Fig. 12), meant that we were limited to optically-transparent media such as air and water. We would also like to note that we did not use experiments to validate the modelled transfer mobilities, or *vice versa*, rather, FEM studies were used to determine the optimal lengths for fabrication of the needles. In terms of practical limitations, the length from tip to needle hub was $\approx$ 0.4 cm longer for the lancet, than the other needles (AX1–3), see Fig. 3b. This could have influenced the modal response of needle construct. In addition, the shape and volume of the soldering at the waveguide-needle termination (see Fig. 3), may have affected the mechanical impedance of the needle construct, introducing uncertainty in mechanical impedance and bending behavior.

To conclude, we have demonstrated experimentally bevel-geometry affects deflection amplitudes in USeFNAB. In the case that higher deflection magnitudes would positively influence the effect of the needle on tissue, e.g. efficacy of post-puncture cutting, the conventional lancet may be recommended for use in USeFNAB, since it achieved the highest deflection magnitude, whilst still maintaining adequate structural rigidity at the tip. In addition, greater tip deflections could enhance bioeffects, e.g. cavitation, as suggested by a recent study^35^, which could be helpful in the development of applications for minimally invasive surgical interventions. Considering that it has already been shown that increasing the total acoustic power could increase biopsy yield in USeFNAB^13^, further quantitative study on sample yield and quality is needed, to assess the detailed clinical benefits of the studied needle geometries.

## Supplementary Information

Supplementary Video 1.

## Data Availability

The datasets produced during this study are available on reasonable request.

## Code availability

The codes used for this study are available on request.
